# Healthy Aging and Dietary Patterns

**DOI:** 10.3390/nu14040889

**Published:** 2022-02-20

**Authors:** Ligia J. Dominguez, Nicola Veronese, Eleonora Baiamonte, Martina Guarrera, Angela Parisi, Chiara Ruffolo, Federica Tagliaferri, Mario Barbagallo

**Affiliations:** 1 Geriatric Unit, Department of Medicine, University of Palermo, 90100 Palermo, Italy; nicola.veronese@unipa.it (N.V.); eleonora_baiamonte@libero.it (E.B.); martiguarrera@gmail.com (M.G.); angelaparisi89@libero.it (A.P.); chiara.ruffolo@edu.unife.it (C.R.); federica.tagliaferri90@gmail.com (F.T.); mario.barbagallo@unipa.it (M.B.); 2 Faculty of Medicine and Surgery, University of Enna “Kore”, 94100 Enna, Italy

**Keywords:** aging, healthy aging, dietary pattern, longevity, diet, lifestyle, chronic, morbidity, mortality, cardiovascular

## Abstract

A number of factors contribute to the complex process of aging, which finally define whether someone will or not develop age-associated chronic diseases in late life. These determinants comprise genetic susceptibility as well as various behavioral, environmental, and dietary factors, all of which have been shown to influence specific pathways regulating the aging process and the extension of life, which makes longevity a multidimensional phenomenon. Although a “miraculous elixir” or a “nutrition pill” are not plausible, researchers agree on the notion that nutritional factors have major impact on the risk of age-associated chronic non-communicable diseases and mortality. In recent years nutrition research in relation to health outcomes has considerably changed from focusing exclusively on single nutrients to considering combinations of foods rather than nutrients in isolation. Although research on specific nutrients is scientifically valid providing crucial evidence on the mechanisms by which nutrition impacts health, the recent switch targeting the multifaceted synergistic interplay among nutrients, other dietary constituents, and whole foods, has promoted emerging interest on the actions of total dietary patterns. This narrative review aims to describe some specific dietary patterns with evidence of associations with reduction in the incidence of chronic diseases allowing older adults to live a long-lasting and healthier life, and confirming the powerful impact nutrition can exert on healthy aging.

## 1. Introduction

Populations around the world are aging at a faster pace than ever in the past and this demographic transition will have a major impact on almost all aspects of society. The achievement of longer life expectancy is a brilliant triumph of humanity, which mirrors improvements in economic and social conditions, as well as outstanding medical progress, leading to reduced maternal mortality, fatal infantile illness, and mortality at older ages [1,2]. This current scenario is illustrated by the fact that life expectancy at the end of the 19th century was similar to that of ancient times when the Roman Empire reigned [3]. On the contrary, European and Japanese populations currently may expect to live over 80 years, while worldwide most people now can expect to live over 60 years [1,2]. Moreover, a 60-year old person today can anticipate living for another 22 years. Worldwide, the average life expectancy at birth rose by 6.2 years, from 65.3 years in 1990 to 71.5 years in 2013 [4]. A study by Foreman et al. reported analyses and prospective data up to 2040 providing a robust, flexible forecasting platform proposing alternative health scenarios in relation to various independent determinants of health, including life expectancy, years of life lost, and a wide range of mortality causes in 195 countries. These analyses found that the projected global life expectancy will increase by 4.4 years (95% UI 2.2–6.4) for men and 4.4 years (2.1–6.4) for women by 2040 to 74.3 years (72.1–76.4) and 79.7 (77.4–81.8), respectively, with variations according to a better or worse health scenarios [2].

Prolonging life expectancy has been accompanied by increasing chronic degenerative conditions that are more frequently observed in older populations, the so called non-communicable diseases (NCDs), such as cardiovascular disease (CVD), neurodegenerative disease, cancer, and diabetes, which account for near 70% of global mortality annually, comprising early deaths (occurring at 30 to 70 years) [4]. This apparent dilemma is predictable, because a longer exposure time to risk factors increases the chances of provoking such conditions. The rise in NCDs occurs worldwide and not only in industrialized nations; indeed, the phenomenon is particularly increasing in absolute numbers in developing and underdeveloped areas because they house most of the world’s population [5,6].

Morbidity and mortality attributable to NCDs have continuously augmented and continues to grow worldwide. Disability and worsening quality of life related to NCDs are also incessantly rising as far as aging of the world population persists [4]. This implies a profound impact on health, health systems, their workforce, and finances [1]. Thus, medicine ought not only to emphasize life extension but especially promote aging as free as possible of morbidity and disability. A key determinant of this alarming scenario has been identified in unhealthy lifestyles of entire populations, which involve a large incidence of risk factors. Overeating unhealthy diets, sedentary behavior, and cigarette smoking will account for the increased incidence of obesity, diabetes, altered lipid metabolism, and hypertension, which are robust CVD risk factors and increase the chances of developing some types of cancer.

Disability-adjusted life years (DALYs), a measure of disability caused by different pathological conditions, were largely attributed to dietary risk factors in 2019: 187.7 million DALYs and 7.9 million deaths. Low intake of fruits and whole grains as well as an elevated intake of sodium were chief dietary risk factors for increasing DALYs and mortality worldwide [7]. Furthermore, nutritional risk factors linked to NCDs escalated considerably from 1990 to 2019. Hence, it is clear that greater endeavors are necessary to promote public awareness and dissemination of dietary practices helping to lessen the health burden linked to unhealthy dietary habits.

For a long time, nutrition research has been concentrated on the health effects of single nutrients. Notwithstanding, people do not eat isolated nutrients but whole foods and combinations of foods; the practice of denoting nutrition as a mere description of nutrients and additional biochemical compounds instead of foods and food combinations is yet prevalent among nutrition researchers, governmental guidelines, food industry, and the general public. That is why many dietary guidelines are limited to recommendations on the intake of nutrients, resulting in substantial implications for science, the general public, and also for industry. Although investigations focused on specific nutrients are valid, as they provide important evidence on the mechanisms by which nutrition impacts health, the food industry and the consumers may misunderstand some of this information, which sometimes may be incorrect or irrelevant. Contrariwise, the recent switch of nutrition research targeting the complex synergistic interplay among nutrients, other food constituents, and whole foods, has encouraged emerging interest on the actions of total dietary patterns [8].

Adherence to a dietary pattern can be assessed using several methods (both, *a priori* as well as *a posteriori)*, which consider combined diverse components rather than only single nutrients or foods. These methods include: the sum of foods/nutrients intake that follow the guidelines recommendations; reduced rank regression, describing linear food intakes arrangements that most likely elucidate midstream markers of disease; and data-driven approaches (e.g., cluster and principal components analyses) defining population actual dietary intake patterns. Altogether, the emphasis on dietary patterns has demonstrated favorable associations of prudent and healthy dietary models, as well as associations with disease risk of a western type diet or dietary patterns rich in meat and refined grains. Even if, owing to the numerous combinations and interactions in a dietary pattern, there is no single optimal approach for describing them, the above-mentioned methods have demonstrated their value in enabling the identification of dietary models more probably associated with healthy aging, such as those described in this review. In addition, the study of dietary pattern may help decisions on nutrition policy, showing the importance of dietary factors in health promotion. Nevertheless, there are still major challenges, for example, how to increase the availability of high-quality food to most of the population while also being sustainable [9].

This narrative review describes certain dietary patterns with epidemiological and clinical evidence of being associated with increased life expectancy, and with a reduced risk of the occurrence of NCDs, allowing people to live a long-lasting and healthier life. We begin with the description of what is today considered a healthy dietary pattern followed by studies on vegetarian, Mediterranean, Japanese, Okinawan, and Nordic diets. Finally, we briefly review the only method that has demonstrated experimentally how to prolong life span by combining different foods: caloric restriction. These dietary patterns confirm the powerful impact of nutrition on healthy aging.

## 2. What Is a Healthy Dietary Pattern?

A dietary pattern indicates all foods and beverages a person habitually eats and drinks, in which components act synergistically to affect health. Hence, dietary patterns may better predict overall health status and disease risk than individual nutrients or foods [8].

According to the Dietary Guidelines for Americans (DGA) 2020–2025, “a healthy dietary pattern consists of nutrient-dense forms of foods and beverages across all food groups, in recommended amounts, and within calorie limits. A healthy dietary pattern can impact health at any stage in life and achieving a healthy dietary pattern at each life stage not only supports health at that point in time, but also supports health in the next life stage and possibly for future generations. If healthy dietary patterns can be established early in life and sustained thereafter, the impact on health could be significant. Establishing and maintaining a healthy dietary pattern can help minimize diet-related chronic disease risk. Conversely, consuming foods and beverages that are not nutrient-dense may lead to disease expression in later years. High intakes of such foods (i.e., an unhealthy dietary pattern) throughout the lifespan can increase the risk of developing chronic diseases”.

Thus, a healthy dietary pattern is one that provides macronutrients in sufficient amounts to meet physiological and energy needs, without excess, while also supply enough hydration and micronutrients to support the physiological body functions. Carbohydrates, fats, and proteins are macronutrients, which make available the energy for cellular processes involved in daily functioning of all the organs in the body. Minerals and vitamins are essential micronutrients notwithstanding the need of small quantities for proper growth, development, metabolism, and general body physiological functions. The definition of healthy dietary patterns is crucial for the formulation of dietary guidance to the general public, for assessment and counselling in clinical settings, for developing practices and policies to improve diet, and to monitor trends in diet quality individually or at a population level. Nevertheless, defining a global healthy diet is not simple because the nutritional needs vary substantially with age, sex, disease status, and physical activity levels, as well as with the cultural context [9].

Carbohydrates are a major source of energy and are mainly supplied by the consumption of grains, fruits, vegetables, and legumes. It is recommended that whole grains be chosen over refined grains, which lose the germ and bran during the milling process resulting in a significantly lower content of micronutrients and fiber [10]. Various meta-analyses of prospective cohort studies have reported associations of whole-grain consumption with a lower risk of ischemic heart disease (IHD), stroke, CVD, and cancer, as well as to reduced total mortality risk and mortality due to CVD, cancer, respiratory diseases, diabetes, and infectious diseases [10,11,12].

Fresh fruits and vegetables provide energy and fiber, which promotes satiety and has beneficial actions on gastrointestinal functioning, glycemic control, and blood lipid levels [13]. Fresh vegetables and fruits are also fundamental sources of phytochemicals, such as polyphenols, carotenoids, and phytosterols, among others. These are bioactive compounds conceivably contributing to the numerous health benefits linked to the frequent consumption of vegetables and fruits [14]. The mechanisms of these benefits are not completely clear, but it has been suggested to be linked to their antioxidant properties, as well as their role in the regulation of fat metabolism, inflammatory mediators, and nuclear transcription factors. For example, flavonoids, a type of polyphenols, have been shown to reduce insulin resistance that may help prevent diabetes [15]. Moreover, polyphenols interact with the gut microbiota enhancing some types of favorable bacteria; in turn, polyphenols are further metabolized by these “good” bacteria to generate additional bioactive compounds [15,16].

A high consumption of fruits and vegetables was reported to inversely correlate with the risk of NCDs, comprising CVD [17,18], hypertension [19], T2D and gestational diabetes [20,21,22], metabolic syndrome [23], chronic obstructive pulmonary disease [24], and colon [25,26,27] and lung cancer [28]. A high consumption of dietary fiber has been consistently related to a reduced all-cause and cause-specific mortality [29,30,31].

Dietary proteins supply energy and amino acids, including essential amino acids, which the human body requires for proper functioning but cannot produce on its own. Proteins can come from animal (meat, eggs, fish, dairy) and plant (legumes, nuts, grains, seeds) sources. Animal proteins have been indicated as richer owing to the content of amino acids, greater bioavailability, and easier digestion [32]. Nevertheless, red and processed meat have been linked to increased mortality, CVD, and some types of cancer [33,34,35,36,37]. In addition, a high intake of heme-iron from meat may lead to endogenous formation of n-nitroso compounds, which have been linked to the development of colon cancer [38,39]. Meat also contains polycyclic aromatic hydrocarbons and heterocyclic aromatic amines, two groups of compounds recognized as carcinogenic [39]. Indeed, consumption of red meat has been associated with an increased risk of diverse types of cancers, including breast, endometrial, colorectal, colon, rectal, lung, and hepatocellular, while highly processed meat consumption has been linked to a higher risk of developing breast, colorectal, and lung cancers [34], as well as increased mortality [35,40,41,42]. In addition, proteins from animal sources increase the dietary acid load, triggering acid-base balance towards acidosis, which has been associated with insulin resistance, impaired glucose homeostasis, and with a higher risk of urolithiasis [43,44].

Consuming an adequate amount of protein is essential to maintain muscle mass and function throughout life. Sarcopenia (loss of muscle mass and function) is more frequently manifested and severe in older adults [45,46,47] and it is also linked to an increased risk of falls and fragility fractures [46,47,48]. Multiple factors concur to increase the risk of malnutrition, especially insufficient protein intake, in older adults [49], including loss of the sense of taste that is common among older people with multimorbidity [50]. For older adults who cannot obtain adequate amounts of dietary protein, supplementation with amino acids can help enhance strength and functional status [51].

Cellular membranes are made of fats as a primary structural component; they are also key energy sources. Dietary fat is generally supplied by an admixture of different types of fats: monounsaturated, polyunsaturated, saturated, and trans fats. Sources of unsaturated fats include plant-derived oils, fish, nuts, and seeds, while animal products (and some vegetable oils) contribute to dietary saturated fats [52]. Trans fats result mainly from processing vegetable oils (making them solid by a chemical process) but are also present in minimal amounts in some animal products [53]. Unsaturated fats have been associated with lower risk of CV mortality, while trans fats and, to a lesser degree, saturated fats have been related to several negative health outcomes, including an increased risk of mortality [52,53,54]. There are two forms of polyunsaturated fatty acids (PUFA) that are essential (i.e., they are necessary for proper growth and reproduction but the body does not produce them): omega-3 and omega-6. Hence, they must be supplied by the diet [55]. Eicosapentaenoic acid (EPA) and docosahexaenoic acid (DHA), two omega-3 fatty acids, have been extensively studied with some evidence indicating cardio-protective effects, prevention of cognitive decline, reduction in inflammation, improving systemic insulin resistance, and sustaining muscle mass [56,57,58]. There are also negative or neutral results, in particular for cognitive outcomes [59]. The main source of EPA and DHA is fish, while widely available supplements may help persons not able to meet their recommended intake with dietary sources [60]. Nuts and seeds as well as vegetable oils supply alpha-linolenic acid, the main plant-derived omega-3 fatty acid [61].

An adequate supply of minerals and vitamins is necessary for proper growth, metabolism, cellular integrity, and general physiological functions. The transformation of diets from rich in whole foods to plenty of refined, processed, and ultra-processed foods has significantly reduced minerals, vitamins, and fiber content in the widely used western diet [62,63]. Cellular aging and late-onset disease have been associated with inadequacies of dietary vitamins and minerals, because scarcity of these key nutrients drives chronic metabolic disruption. Thus, adequate dietary intake or supplementation of nutrients with potential antioxidant properties (e.g., vitamins E, C, A, as well as copper, magnesium selenium, and zinc) has been proposed as a strategy to lessen the risk and progression of age-associated NCDs [64].

The human body is mainly composed of water; it makes up most of lean body mass and body weight. Water is not only a source of hydration but also of micronutrients, comprising electrolytes, and trace elements [65]. Drinking water can provide as much as twenty percent of the calcium and magnesium daily recommended intake. Unfortunately, the knowledge on water requirements and actions on health and disease is limited. Nevertheless, the worrying global escalation in the consumption of high-calorie beverages (e.g., sugar-sweetened beverages) has redirected the attention on the importance of drinking water for maintaining health and preventing disease [65].

In summary, based on current knowledge of nutritional requirements and their expected impact on health outcomes, healthy dietary patterns can be defined as those that are rich in health-promoting foods, such as vegetables, fruits, whole grains, legumes, nuts, and vegetable oils, includes a low to moderate amount of seafood and poultry, includes low quantity of red meat, processed meat, added sugar, refined grains, and starchy vegetables, and do not include trans fats [9]. In some world regions, these healthy dietary patterns have been occurring naturally as part of their local history, traditions, and food sources, as in the case of the traditional Mediterranean diet (Med Diet) and Japanese/Okinawan diets. Some other healthy dietary patterns have been developed based on the evidence that some combinations of foods/nutrients may be protective for some health outcomes (e.g., the dietary approaches to stop hypertension (DASH) [66] and Med Diet-DASH Intervention for Neurodegenerative Delay (MIND)) [67]) that share some common features.

## 3. Vegetarian Diet

The reasons for adopting a vegetarian dietary pattern are generally following health, environmental, ethical, religious, cultural, political, economic, esthetic, and culinary inclinations. Overall, these types of diets are not characteristic of specific geographical locations or ethnic groups (although in some nations there are many people who follow vegetarian diets), but they refer to the total or partial exclusion of foods derived from animal sources. Vegetarian diets comprise an abundant and varied inclusion of plant-based foods, such as vegetables, fruits, nuts, grains, seeds, herbs, spices, mushrooms, as well as vegetable oils. The different variations of vegetarian diets comprise: (1) vegan (exclusion of all animal products including honey); (2) raw vegan (same as vegan but using a temperature below 48 °C for cooking); (3) lacto-vegetarian (with dairy products but excluding eggs); (4) ovo-vegetarian (with eggs and excluding dairy products); (5) lacto-ovo vegetarian (with both, dairy products and eggs); (6) pesco-vegetarian (with fish and seafood). All these variations withhold the consumption of animal meat (besides fish in pesco-vegetarian) and may abstain from animal slaughter by-products (i.e., gelatin and rennet, a complex set of enzymes produced in the stomachs of ruminant mammals used in the production of cheese) [68]. It is worth mentioning that lately vegetarian diets have become more popular not only for their eventual health effects but also because they favor a lower environmental impact. In fact, a diet based on plant-derived foods produces substantially lower emissions of greenhouse gas vs. animal-based diets, hence decreasing the environmental damage [9,69,70]. However, a cause of concern in following a strict vegetarian diet is the increased risk of nutritional deficits, i.e., vitamin B12, omega-3 PUFA, vitamin D, protein, calcium, iron, and zinc [71,72].

One of the “Blue Zones”, that is, geographical areas with the highest concentration of centenarians in the world described by Poulain et al. [73], is located in Loma Linda (California, USA), where a population of about 9000 people belonging to the Seventh Day Adventist (SDA) community appears to live longer and have reduced incidence of cancer and CVD compared to other people from California. Analyses of data from the Adventist Health Study 2 (AHS-2) reported that after a follow-up of 5.79 years those participants adhering more closely to all types of vegetarian diet had a lower risk for total mortality. Yet, in analyses separating the different types of vegetarianism only pesco-vegetarians exhibited reduced incident mortality, whereas the other types had no benefit. In addition, the associations in men were more often significant vs. those in women [74]. More recent analyses, comprising other prospective studies besides the AHS, reported significant but smaller risk reductions for cerebrovascular disease, CVD mortality, chronic kidney disease, and type 2 diabetes (T2D) [72]. A systematic review comprising 86 cross-sectional and 10 cohort studies reported a reduction in body mass index (BMI), total and low density lipoprotein (LDL)-cholesterol, as well as blood glucose levels in vegans and vegetarians vs. omnivores in studies with cross-sectional design. In addition, the authors observed a significant reduction (−25%) in the risk of IHD incidence and/or mortality, and of incident cancer (−8%) in prospective studies, without changes in total mortality or mortality due to cerebrovascular disease, all CVD, and cancer. Taking into consideration only vegans, the authors observed a reduced risk in the incidence of total cancer (−15%); however, the number of studies was inadequate [75]. A meta-analysis of eight studies comprising 183,321 participants showed substantial heterogeneity, in particular among studies of SDA cohorts. Indeed, SDA studies found larger sizes of the effects vs. studies in populations different from SDA cohorts that reported significance only for the reduction in IHD. Thus, the conclusion was that a vegetarian diet was associated with a modest CV benefit, but no clear reduction in total mortality. Moreover, the benefit was mainly attributable to SDA studies, while the effects in other cohorts remained unproven [76]. It is crucial to consider that Adventists have other healthy lifestyle behaviors, comprising regular exercise and no smoking, which can enhance the protection against NCDs. A recently published study compared AHS-2 participants with a group of non-smokers from the US populations and reported lower total and cancer-related mortality, as well as lower incident breast, lung, and colorectal in AHS-2 participants compared to controls [77]. Former studies using data from the AHS-2 (*n* = 125,000) concluded that a vegetarian diet has likely protective effects against the risk of developing obesity, T2D, and metabolic syndrome [78,79]. Nevertheless, it is still not completely clear if the reduced incidence of T2D in vegetarians may be attributable to the avoidance of meat or to the prominent consumption of plant foods. Regarding dementia, a former small analysis of a sample from the AHS found that participants eating meat had a double risk of developing dementia when compared to vegetarians. A second analysis in a larger sample did not observed any difference between vegetarians vs. non-vegetarians on incident dementia. These analyses had important limitations including lack of definition of standardized cognitive assessment during the studies [80]. Because both SDA and Baptist communities follow healthy lifestyles, a study evaluated the effects of lifestyle risk factors on hospital admissions for some neurological diseases among 6532 SDA and 3720 Baptists. There was a lower incidence of dementia for both communities, whereas the incidence of epilepsy and Parkinson’s disease was not different compared to the incidence in the general population [81]. Thus, further investigations are needed to better appreciate the interaction between lifestyle and other components of religious beliefs.

Because the consumption of meat, in particular red and processed meat, has been associated with increased mortality and other harmful health outcomes [34,35,40,41,42], it is challenging to elucidate if the eventual benefit of vegetarian diets stems from the conspicuous consumption of plant foods, the absence of meat consumption, or the replacement of meat by other types of food. Regardless, vegetarian diets continue to be reported as associated with health benefits. Compelling evidence supports the notion that a diet mainly composed of plant-derived whole foods, minimizing the consumption of processed and ultra-processed foods, is a potential general healthy diet. There is a current lack of consensus on a precise definition of vegetarian diets in epidemiological studies, which may help to explain the non-homogenous results. Nevertheless, it is vital to assess and indicate the integration of vitamin B12, vitamin D, and other nutrients when a deficiency of these nutrients is suspected while following a vegetarian diet [68,82,83].

## 4. Mediterranean Diet (Med Diet)

Accumulated evidence, which began with the Seven Countries Studies [84] and continued steadily, has shown that the dietary and lifestyle habits traditionally followed by residents of the Mediterranean basin, may play a key role in the prevention of premature mortality and in reducing the incidence of NCDs [85,86,87,88,89]. In the seminal Seven Countries Studies it became clear for the first time that populations from Southern Europe, specifically from countries where olive-trees are indigenous, had life expectancies among the highest in the world as well as the lowest incidence of IHD, cancer, and other NCDs [84,90]. At the time when Keys et al. uncovered and named it the “Mediterranean diet” in the 1950s, these populations followed traditional food and lifestyle habits, which originated many centuries earlier and had remained similar in the same geographical locations. The uninterrupted permanence of these dietary and lifestyle behaviors has constantly stimulated interest among health researches, gastronomes, agronomists, and historians among other scholars. After that first epidemiological study, many investigations have produced a myriad of data, most of them showing its benefits, in such a way that the Med Diet is the eating model with the largest number of publications in the medical literature compared with other dietary models [89].

The Med Diet is indicated as being among the healthiest recommended dietary patterns in the 2015–2020 DGA [91]; it has also been associated with greater nutrient adequacy, because persons adhering to this dietary model have a low risk of presenting deficiency of fiber, magnesium, calcium, and potassium, all crucial nutrients that have been recognized as relevant public health concerns [92]. The Med Diet is mainly grounded on plant-derived foods but it also admits animal-derived food in low amounts consumed occasionally. In addition, because the Med Diet favors local and seasonal food production and consumption, it is considered an eating model that also respects the environment. It is important to clarify that the traditional Med Diet does not mean only a list of foods that are frequently consumed in countries from the Mediterranean basin. This dietary model also comprises the way foods are grown, selected, minimally processed, and distributed, in conjunction with other grounds of a healthy lifestyle.

The Med Diet involves the regular consumption of colorful varied and seasonal vegetables daily with none or minimal gastronomic interventions; seasonal fresh fruit consumed at the end of every meal as dessert; inclusion of nuts and seeds in the traditional recipes or as snacks; various types of legumes consumed several times per week; unprocessed grains (bread, rice, pasta) mostly whole consumed every day; cold pressed extra virgin olive oil consumed daily (chief source of dietary fat, for cooking and seasoning); addition of herbs and spices for flavoring; consumption of fish in moderate amounts (2 to 3 times per week); daily consumption of dairy products, i.e., milk and yogurt, and small portions of cheese occasionally; consumption of eggs as sources of high-grade proteins (2 to 4 weekly); occasional consumption of sweets, cakes, and dairy desserts in small amounts (few times per week); red and processed meat consumption in small portions and not frequently (once to twice monthly); drinking plenty of water as the main beverage; consumption of wine in moderation (for women ≤1drink/day and 1 to 2 drinks/day for men) always with meals respecting former habits and beliefs of each community [88]. The Med Diet is composed chiefly of unprocessed foods rich in nutrients (nutrient-dense), as opposed to “empty calories” of westernized diets, which are full of processed food (rich in calories but poor in nutrients), unquestionably linked to the high risk of obesity and NCDs. The Med Diet also comprises other non-nutritional components mentioned above as well as having an adequate rest (nighttime sleeping of sufficient duration and quality with short daytime periods of sleep if needed [siesta]) [89]. The term Med Diet, besides its value from a nutritional point of view, has insightful significance related to a unique lifestyle of populations living in the Mediterranean basin. This includes knowledge, abilities, practices, and traditions, transmitted from generation to generation, comprising traditional cuisines rich in colors and aromas, emphasizing the connection with the natural environment and the importance of preparing and consuming meals together with family and friends [89,93]. At the time of the Seven Countries Study the production of food was not industrial and involved conspicuous physical effort, essential to further explain the multiple benefits resulting from merging dietary and other lifestyle factors [94]. All these features were the reasons why UNESCO recognized the Med Diet as intangible cultural heritage of humanity in 2010 [93].

Unfortunately, a large portion of the population that today lives in the countries around the Mediterranean basin, where the beneficial effects of this paradigm of healthy eating and lifestyle were first observed, do not follow this dietary pattern, primarily for portion sizes (which are always larger than they used to be), proportions of food groups (with inclusion of frequent consumption of red and processed meat, among others), and frequent use of processed industrial food [95]. It is worrying to note how the most affected by this phenomenon of abandonment of the traditional Med Diet and lifestyle are children and adolescents [96], probably owing to the pervasive dissemination of globalization and westernization of the production and consumption of food, which is currently shaping and homogenizing the eating behavior worldwide [9].

A large body of medical literature shows that a higher adherence to the Med Diet has been associated with a reduced risk of total mortality [97,98]; reduced incidence of CVD and related mortality [86]; reduced cognitive decline, dementia, and depression [59,99,100,101]; reduced mortality due to cancer and lower incidence of colorectal, breast, gastric, respiratory, bladder, liver, and head and neck cancer [102]; weight loss and reduction in BMI [103]; decreased incidence of T2D [104]; reduced risk of chronic obstructive respiratory disease [105]; decreased incidence of frailty [106,107,108,109,110,111,112]; as well as reduced incidence of fragility fractures [113,114,115]. Morris et al. created a combination of components of the Med Diet and DASH with greater evidence of neuroprotective effects in a dietary model known as MIND diet [67]. These dietary patterns include foods with B vitamins, polyphenols and other antioxidants, monounsaturated fatty acids (MUFAs), PUFAs, fiber and DHA, which may help to explain their neuroprotective actions. The Med, DASH, and MIND diets are mainly based on foods with plant origins and all these models recommend consuming red meat, sweets, processed/ultra-processed foods, and sugar-sweetened beverages with frugality and low frequency. These foods are usual in western dietary patterns often associated with harmful health outcomes.

In addition to its numerous health benefits, the Med Diet highlights following the seasonal crop cycles, attentiveness to biodiversity, guaranteeing proper balance among human beings, nature, and renewal of resources [93]. Due to all these reasons, the Med Diet is considered a healthy dietary and lifestyle model, confirmed to be one of the most sustainable nutritional patterns for both our health and the environment.

## 5. Japanese Diet

Japan has several records: it is the country with the longest life expectancy, the greatest number of centenarians, and the most accelerated growth of aging in the world [116]. Life expectancy in Japanese women increased at a steady rate of near 3 months every year for the previous 160 years in 2002 [117]. Life expectancy of Japanese women in 2016 was 87.1 years, the longest among G7 countries [118]. Japan is composed of more than 3000 islands with various historical, religious, and cultural influences and contacts with other nations, which have created the unique and peculiar Japanese diet. Other healthy lifestyle and social components of the Japanese way of living may as well help to explain Japan’s outstanding longevity achieved in a relatively short period of time and linked to a fast reduction in NCDs-related mortality in the 1950s and 1960s; this was followed by a large decline in stroke-related mortality rates, a chief cause of mortality in the 1950s. After the mid-1960s, gains in health were sustained through the implementation of primary and secondary preventive community public health programs, together with the use of cutting-edge medical technologies and a total coverage insurance system. This also involved lessening inequalities by the provision of educational opportunities and financial access to the population. At present, accelerated population aging embraces new major challenges to support the health improvements of the aging population [116].

The record longevity of the Japanese population has aroused great interest in the scientific community in order to elucidate the possible contributing factors, first of all their dietary traditions. The Japanese diet is mostly composed of small portions of traditional seasonal foods (rice, vegetables, fish, soy, green tea, and seaweeds), together with the practice of “*hara hachi bu*”, a traditional custom denoting that a person should eat until he/she is 80% full [119]. Carbohydrates provide 60–65% of total calories, mainly from rice; fats are responsible for 20–25% of total energy; whereas proteins (mainly derived from plants) account for the remaining 5–10% of calories. Japanese consume a lower amount of fats compared to Med Diet and the ratio of omega-6/omega-3 is around 2 to 3 [120].

In 1980, the Japanese Ministry of Agriculture, Forestry, and Fisheries proposed for the first time the concept of a “Japanese Diet”, identifying the health advantages compared to western diets, a notion already recognized by the Seven Countries Study [90]. Subsequently, in 2005, the Japanese government started several dietary recommendations programs representing the traditional Japanese diet as an inverted food pyramid, evocative of a spinning top. Running people on the spinning top emphasized the relevance of regular physical activity and drinking water. Four layers were allocated in the inverted cone: the highest consisted of dishes containing grains (i.e., rice, noodles); the following layer comprised plant-based dishes (i.e., vegetable and soups); the third layer was composed of fish, meat, eggs, and soy-bean dishes; the fourth layer included fruit and dairy products [121]. Conformance with the Japanese Food Guide Spinning Top was examined in relation with total and cause specific mortality among 36,624 participants aged 45–75 years recruited from eleven public health centers across Japan. Better adherence to the food recommendations was associated with significant reduced total and CVD-related mortality, and lower incident cerebrovascular disease. The authors reported some evidence, yet not significant, of an inverse association with cancer mortality [122]. In accordance with these results, obesity-related cancer onset was lower in the Japanese population vs. the USA population [123].

As mentioned, a sample of the Japanese population was also included in the Seven Countries Studies; together with the Med Diet, the Japanese diet was associated with a reduced incidence of IHD and all-cause mortality vs. samples from Finland and the USA [90,124]. More recent results from observational studies have corroborated these findings, showing that the Japanese diet is associated with a reduced risk of CV mortality [125,126]. Okada et al. created a Japanese diet score (JDS) including seven food categories (vegetables, fresh fish, Japanese pickles, beans and bean products, seaweeds, fruits, and fungi). The authors observed that a higher score of the JDS was associated with a reduction in total and CVD mortality, particularly in women [126]. A possible explanation of this association may be the elevated intake of omega-3 PUFA [127].

A study evaluating the association of adherence to the Japanese dietary pattern and disability-free survival (DFS) among 9456 Japanese older adults, with a 10-year follow-up used a validated Japanese Diet Index (JDI). Results showed that a higher JDI was associated with a longer DFS time vs. the lowest JDI score. Each 1-SD increase in JDI score was associated with 3.7 months of life added without disability for men and women regardless of baseline chronic conditions [128]. Concerning cognition, a study including 1006 community-dwelling Japanese people aged 60–79 years and followed up for a median of 15 years reported that a high consumption of vegetables, seaweeds, soybeans and derivatives, fruit, fish and a low rice consumption was associated with a lower risk of Alzheimer’s disease [129].

There are also some negative aspects of dietary patterns in Japan. A widespread habit in Japan, as well as in other eastern Asian countries, is an elevated intake of salt. It is well-known that a diet with excess salt is associated with a higher risk of hypertension, and maybe with a higher incidence of gastric cancer [130]. As such, in highly populated and industrialized metropolitan areas of Japan there is a high risk of stroke and cerebrovascular disease; populations living in these areas have excessive consumption of salt, as well as high rates of smoking, stress, and alcohol abuse, which may help explain the elevated prevalence of hypertension and consequent cerebrovascular disease in these locations [125,131]. In fact, the current lifestyle of Japanese has acquired many western habits. Kanauchi et al. developed a 12-item Japanese diet score (JDS) that could be self-administered to evaluate adherence to this dietary pattern in 1458 Japanese participants aged 18–84 years. Almost half (47.7%) reported a low JDS score with only 11.1% reporting a high adherence. Participants who were younger, heavy drinkers, and physically inactive had lower adherence to JDS after multivariate adjustments [132]. Therefore, a Japanese diet pyramid comparable to the Med Diet pyramid was proposed as an instrument for nutrition education.

Nevertheless, the past higher prevalence of gastric cancer in Japan (without counting Okinawa) vs. North American and European countries, linked to the high salt consumption and to the frequent use of salt preserved foods [133], has progressively improved in recent years attributable to the recommendations of avoiding excess consumption of salt and adopting healthy eating [134].

Taken together, these findings suggest that the traditional Japanese dietary pattern can be considered one of the key factors contributing to the notable longevity of this population compared to western nations. The fact that Japan has also been turning towards westernization of diet and lifestyle in recent decades highlights the urgent need for promoting the traditional diet among the population to maintain high standards of longevity and healthy aging.

## 6. Okinawan Diet

Among the Japanese, who already have the longest life expectancy in the world, there is a region that is home to the population with the greatest number of centenarians worldwide: the Okinawa prefecture, which exceed even the rest of the Japanese in life expectancy. According to data from the Japan Ministry of Health, Labor, and Welfare, Okinawan women at birth could expect to live 87.02 years vs. 86.35 for all Japanese women (average from 47 prefectures) in 2013; Okinawan men at birth could expect to live 79.40 years vs. 79.59 years for all Japanese men [135].

The diet followed in Okinawa by these long-lived populations is attractive because they not only live longer but most of them maintain active lives. In fact, Okinawa is part of the five “Blue Zones”, which comprise populations with the world’s longest-lived people and the lowest risk of age-associated diseases [73]. Okinawa is the largest of the Japanese Ryukyu Islands, where the traditional diet is very rich in nutrients and low in calories. Due to the exceptional and healthy longevity of its inhabitants, the Okinawan diet has aroused particular interest in recent years [136]. Various studies have intensely examined the components of the Okinawan diet in order to explain their long life expectancy and, above all, their lengthy health span. However, life expectancy has decreased lately in Okinawa, probably due to a reduction in the adherence to the traditional local diet, together with other possible genetic, lifestyle, and environmental factors.

The Okinawan diet is composed mainly of plant origin foods with 80% of its calories coming from vegetable sources, specifically, whole grains, fruits, legumes (mainly soy), relatively small amounts of fish, and limited amounts of lean meats [137]. The traditional Okinawan diet has 20% less calories compared to the typical Japanese diet, and a large proportion of colored vegetables. Sweet potatoes *(Ipomoea batatas)*, the main source of carbohydrates in the Okinawan diet, are rich in antioxidants and have a low-glycemic index, while the main source of carbohydrates in the Japanese diet is rice. The Okinawan diet has low amounts of saturated fats derived from a limited consumption of eggs, meat, and dairy products. Interestingly, Okinawan people follow a natural example of caloric restriction with optimal nutrition, suggested to be a potential modifier of longevity [138,139]. Okinawan centenarians follow the Confucian teaching of “*hara hachi bu*” [119], which is in fact a sort of caloric restriction.

Among Okinawans, the incidence of prostate, colon, and breast cancers is about 50% lower vs. the rest of Japan [137], which has been largely attributed to the scarce intake of saturated fats, the large quantities of antioxidant and anti-inflammatory compounds taken from the diet, and the low omega-6/omega-3 ratio [127,137]. Although stroke and cerebrovascular disease risk is elevated among Japanese people living in metropolitan areas who use the traditional diet and lifestyle less [125,131], people from Okinawan have a further reduced risk of IHD, stroke, and cerebrovascular disease [137,138,140]. Obesity prevalence is only 3–4% in Japan, much lower vs. the prevalence in Europe and in the USA, where obesity largely contributes to the high prevalence of NCDs. Obesity and T2D are also rare among centenarians from Okinawa [138].

## 7. Nordic Diet

The Nordic diet has been proposed as a model representing foods consumed in five Nordic countries: Denmark, Finland, Iceland, Norway, and Sweden, where the typical dietary habits comprise a high consumption of fish, cabbages, root vegetables, pears, apples, berries, whole grains, potatoes, low-fat dairy products, and rapeseed oil [141]. The so-called New Nordic Diet (NND) was developed in Copenhagen as a paradigm of healthy and sustainable diet integrating dietary and gastronomic traditions from the five mentioned Nordic countries. The NND concept received the input from experts in human nutrition, gastronomy, environmental issues, food culture, history, and sensory science, and also from experts with knowledge about children and their food habits and preferences as part of a multidisciplinary 5-year research project “Optimal well-being, development and health for Danish children through a healthy New Nordic Diet” (OPUS), which aimed to define and test the NND [142].

Interestingly, NND has several components that are also part of the Med Diet and Japanese diet (Table 1). For example, it includes organically and local grown fruits (i.e., berries, pears and apples), vegetables (i.e., cruciferous vegetables, wild aromatic herbs, edible plant roots, and green leafy vegetables), nuts, seeds, legumes, whole grains (i.e., rye, oats and barley), fish (i.e., salmon, sardines, mackerel, herring), herbs, seaweeds, and mushrooms; NND contains low amounts of meat, sweets and fat, and avoids processed food [142,143]. Hence, NND also follows principles of environmental protection and sustainability as the Med Diet. The main difference between the two diets is the primary fat source, which is extra virgin olive oil in the Med Diet and rapeseed/canola oil in NND. As mentioned above, an extensive medical literature supports the Med Diet as a way to prevent NCDs; the NND still needs more such studies because some current results are still conflicting. More longitudinal and large prospective studies are needed in the future to provide further evidence-based recommendations [143]. The key messages underlying the NND guidelines are the following: (1) including more calories from plant-based foods and less from meat; (2) use of more foods coming from the lakes and sea (comprising seaweeds and shellfish); and (3) including more wild local foods from the countryside (plants, berries, mushrooms, and aromatic herbs) [142].

Although NND has been developed in recent times, the literature on this healthy dietary pattern has grown, showing significant associations between NND and several health outcomes including various NCDs (i.e., CVD, obesity, some types of cancer, neurodegenerative diseases) and mortality [59,141,144,145,146,147,148,149,150,151,152,153,154,155,156,157,158,159], thus increasing overall life and health expectancies.

Several clinical trials from Nordic European populations showed that healthy Nordic dietary patterns determined a reduction in CVD risk factors with a similar extend as previously reported for the Med Diet [145]. In particular, following the Nordic diet was associated with improvements in blood lipid profiles [146], lower blood pressure in patients with hypercholesterolemia [147], reduction in diastolic and mean blood pressure in persons with features of the cardiometabolic syndrome without changes in body weight [148], and reduction in blood pressure in persons with central obesity [149]. However, in a recent study conducted in 1981, men aged 42–60 years and free of coronary heart disease (CHD) at baseline in 1984–1989 reported no association of adherence to a healthy Nordic diet with a lower risk of CHD or with carotid atherosclerosis or major CHD risk factors, except for an inverse association with serum C-reactive protein concentrations [150].

The NND has shown some effects on the control of overweight. A 6-month clinical trial conducted in Denmark in a controlled, free-living setting and involving obese participants (*n* = 64; mean age 44 years) showed that NND compared to an average Danish diet was associated with an improvement in insulin resistance index, which provides the possibility of using NND as a strategy to prevent the onset of T2D in obese persons at risk [151]. Similarly, *ad libitum* NND produced weight loss in centrally obese participants [149]. Whole grains, a typical food in the NND, showed a likely protective effect against the development of T2D [144]. A recent study in middle-aged and older men from eastern Finland (*n* = 2332; aged 42–60 years) free of T2D at baseline reported that after an average follow-up of 19.3 years the lowest vs. the highest quartile of adherence to NND was associated with a higher risk of incident T2D and with a higher blood glucose and insulin concentrations after adjustment for confounders [152]. In the same cohort, participants with higher adherence to NND exhibited a 9% reduced incidence of colorectal cancer (CRC) [153]. In accordance with these findings, the consumption of NND foods rich in fiber were also significantly related to a lower risk of incident colon cancer [144]. Another study showed that after CRC diagnosis, a higher concordance with both the Med Diet and NND was related to a better survival in long-term CRC survivors [154].

Regarding cognition, a study involving participants aged 57–78 years cognitively intact at baseline showed that over a 4-year study period a higher concordance with the Nordic diet was related to better scores in global cognition after adjustment for demographic and lifestyle confounders [155]. Analyses of data from a prospective cohort study (*n* = 2223) in over-60 participants who were cognitively intact at baseline with a mean follow-up of 6 years reported that a moderate to high concordance with the Nordic diet was more significantly related to a lower cognitive decline compared to other healthy dietary models, including the Med Diet [156]. Further analyses of this cohort study showed that the association of Nordic Prudent Dietary Pattern (NPDP) with a reduced decline in Mini Mental State Examination scores was stronger adding moderate to intense physical, mental, or social activities [157].

For stroke prevention, among 55,338 men and women from the Danish Diet, Cancer and Health cohort a higher adherence to a healthy Nordic diet, as reflected by a higher Healthy Nordic Food Index score (4 to 6 points) vs. a low adherence (0 to 1 points), was related to a significantly reduced risk of ischemic stroke during a median follow-up of 13.5 years [158].

The “Diet, Cancer and Health Study”, a 12-year prospective observational study involving 57,053 Danish participants, aged 50–64 years reported a significant reduction in overall mortality in those with a higher adherence to NND evaluated with a Nordic food index (which included rye bread, cabbage, fish, edible plant roots, pears, and apples) compared to the lowest adherence. In an adjusted model, a 1-point higher Nordic index score was associated with about a 4% lower mortality rate in men and women. When the index components were evaluated separately, whole grain rye bread consumption was the factor most consistently associated with lower mortality in men [159].

In summary, the Nordic diet can be defined as another example of regional diet taking health, palatability, food culture, and environmental sustainability into account [142]. Although the Nordic dietary pattern appears promising as a healthy, palatable, and sustainable diet, further research is still needed to confirm its effects in larger well-designed studies from other Nordic populations and also from populations of other countries outside the Scandinavian nations.

## 8. Caloric Restriction with Optimal Nutrition (CRON)

Although caloric restriction (CR) is not in itself an eating pattern, avoiding excess intake of calories can be applied to any diet and is common to dietary patterns that are associated with reducing the risk of NCDs and improved longevity. Indeed, in experimental studies, CR is one of the interventions with more evidence able to extend life expectancy and delay the onset of multiple age-associated diseases in diverse species, from yeast to mammals. In particular, CR refers to the reduction in the total energy intake by 20 to 40% without causing malnutrition or deficiency in essential nutrients. Regardless of being so successful in longevity and health promotion in experimental models, in humans long-term CR has been associated with both beneficial and detrimental effects [160].

Numerous studies conducted over eighty years have shown that dietary restriction prolongs lifespan in various experimental models compared to *ad libitum* feeding. CR has been investigated in humans as well. The first experimental study was published in 1935 by Clive McKay at Cornell University validating this hypothesis in rats with later studies ratifying the original findings in a number of experimental models indicating a net dose-dependent effect between the degree of energy intake reduction and the subsequent extension of median and maximum lifespan [160].

There are two studies performed in nonhuman primates with contradictory results. The first study carried out at the University of Wisconsin-Madison (UWM) found that primates under CR had longer lifespans [161]; whereas the second study conducted by the National Institute of Aging (NIA) did not observe any difference [162]. A plausible explanation for the conflicting results was the diet composition of the control groups: the *ad libitum* diet (control) in the UWM study had a high content of sugar, possibly responsible for a reduced lifespan vs. the CR group. Contrariwise, the *ad libitum* control diet in the NIA study was healthier, which could have made the effects equally beneficial as the CR group. Primates under CR in both studies showed improvements in metabolic profiles and reduction in the formation of amyloid brain plaques [161,162].

Although CR does seem to extend lifespan in all species, it is associated with bone loss and fragility fractures [163]. Species living in constant environments such as laboratories have less chances to trigger mechanisms in response to food restriction [163]. There is excessive availability of food and little opportunity for exercising in the laboratory and a reduction in natural enemies (e.g., infections), rendering laboratory animals tangibly different from wild animals and possibly more responsive to CR [164].

In human studies, as shown also with healthy dietary patterns, CR has shown protective effects on atherosclerosis, improvements in cardiac function, and reduction in overweight and obesity [160], even if the benefit is similar to that obtained by physical exercise. There is no doubt that avoiding an excessive intake of calories is helpful in overweight patients and that a high-calorie diet is harmful for most people being a well-known major CV and metabolic risk factor. However, a true delay of aging or an extension of lifespan with CR in humans is not yet feasible. The benefit of CR in lean people already following a healthy diet and lifestyle is uncertain. Moreover, there are important adverse effects of CR including infertility, chronic lack of energy perception, mental stress, and sexual dysfunction that may result in depression and loss of appetite with increased risk of anorexia [165].

The founders of the “Caloric Restriction Society” proposed the CRON-diet recommending a reduction in total calories of 20% according to individual basal metabolic rates. Remarkably, the Okinawan dietary pattern and lifestyle (Section 6) of dwellers from the Ryukyu Islands, the region with the greatest number of centenarians in the world, may represent a natural model of CRON-diet. The traditional diet from Okinawa has a lower caloric content compared to the traditional Japanese diet, which already has less calories compared to the typical western diet, and contains mostly vegetables (near 80% of calories are of plant origin; Okinawans consume about one kg of vegetables daily comprising seaweed; and 50% of calories come from sweet potatoes), a half serving of fish and legumes per day including soy. Consumption of red meat, meat products, eggs, and dairy is low [138]. The Confucian teaching ‘*hara hachi bu*’ educates people from Okinawa to eat only up to 80% full satiety. Thus, practicing ‘*hara hachi bu*’ renders this long-lived people a unique human population having a self-imposed CR training.

Various mechanisms have been suggested in order to help explain why CR promotes lifespan extension [160]. Autophagy is one of these mechanisms, which refers to the cellular self-digestion involved in degradative processes of proteins and organelles. When cells age, proteins and organelles that have undergone damage are accumulated turning cells into pathogenic phenotypes with mutant proteins that tend to aggregate. These modified constituents are harmful, particularly in non-dividing differentiated cells, i.e., cardiomyocytes and neurons, where functional deterioration manifests as the organism ages. CR is associated with increased autophagy (which is beneficial), possibly because CR reduces insulin and insulin inhibits autophagy. In addition, it has also been proposed that CR reduces oxidative damage, improves insulin sensitivity, and reduces tissue glycation [160,166,167,168]. An interesting mechanism suggested to explain CR effects is hormesis, which affirms that CR denotes a low-level stress that induces the development of greater defenses slowing the aging process [169].

## 9. Conclusions

Nutrition research has considerably changed over the past century. At the dawn of the nutrition field, the focus was on identifying and managing diseases linked to overt nutritional deficits, which has shifted to the new paradigm that concentrates on lifestyle-related diseases, associated with excess caloric intake, sedentary behavior, and stress. Technology and development of food industry, as well as advances in nutrition investigation, have backed the decreasing frequency of diseases linked to nutritional deficit in most regions, concurrently perceiving the growing challenge of increasing rates of obesity, aging, and NCDs. The former reductionist approach, guided by the purpose of understanding cellular and molecular mechanisms explaining the effects of single nutrients has evolved into a more general goal of discerning the role of combinations of foods and nutrients in dietary patterns as crucial foundations of health and disease. In fact, a dietary pattern may better reflect the possible synergistic or antagonistic actions of the different components rather than the action of single components [8]. It should be considered that the eating models are also connected to social, behavioral, geographical, cultural, and environmental factors that powerfully influence preserving health and preventing diseases.

In this narrative review, we examined the features of some dietary patterns that up till now have accrued more evidence on associations with lengthy survival and with reduced incidence of NCDs. People living in the so called “Blue Zones”, world areas where exceptional longevity has been reported, have followed these types of dietary models through life, namely Mediterranean, Japanese, Okinawan, and vegetarian. Some of the characteristics common to these dietary models have been linked to mechanisms suggested to explain healthy aging; for example, the extensive inclusion of foods derived from plants and whole grain products, which have plenty of fiber, minerals, vitamins, and phytonutrients that have shown antioxidant and anti-inflammatory properties; the use of mono- and polyunsaturated fats with potential cardio-protective effects; protein consumption in moderate amounts and mostly plant-based, with the exception of fish consumption; the inclusion of spices and herbs that have shown antioxidant and anti-inflammatory actions; alcohol consumption in moderate amounts or no consumption; an infrequent consumption of red and processed meat or avoiding it; avoiding the consumption of processed/ultra-processed foods and use of only small quantities of sugar, all of which have been reported to be linked to increased risk of obesity, T2D, CVD, and several types of cancer. The favorable dietary components (Table 1) consumed in moderation together with other key determinants (i.e., physical activity, sleep quality) identified in populations that usually follow these dietary patterns, may exert a hormetic positive stress that may help to slow down the aging process [169].

The scientific evidence that has accumulated to date confirms the fundamental role that diet exerts in modifying aging mechanisms and in the development of age-related chronic diseases. Further endeavors are necessary to assimilate these healthy dietary and lifestyle preferences in daily life among communities worldwide in order to render healthy eating widely accessible and sustainable.

## Figures and Tables

**Table 1:** Comparison of the main components of dietary patterns associated with longevity.

	Vegetariana *	Mediterranean *	Japanese *	Okinawan *	Nordic *
**Vegetables**	++++	++++	++++	++++	++++
**Fresh Fruit**	++++	++++	++++	++++	++++
**Nuts and Seeds**	++++	++++	++++	++++	++++
**Legumes**	++++	++++	++++	++++	++++
**Whole Grains**	++++	++++	++++	++++	++++
**Fish**	pesco-vegetarian	+++	++++	++++	++++
**Red/Processed Meat**	-	+	+	+	+
**Poultry**	-	+	+	+	+
**Dairy**	lacto-vegetarian	++	+	+	++
**EVOO**	***	+++	***	***	**
**Wine**	-	++	-	-	-
**Herbs and Spices**	++++	++++	++++	++++	++++

EVOO: extra virgin olive oil. * all these dietary patterns emphasize the use of locally and seasonally produced food. ** rapeseed/canola oil more frequently used. *** vegetable oils, not exclusively EVOO.

## Data Availability

Information on the studies included in the review is available upon reasonable request to the Corresponding Author.
